# Adjacent Habitat Influence on Stink Bug (Hemiptera: Pentatomidae) Densities and the Associated Damage at Field Corn and Soybean Edges

**DOI:** 10.1371/journal.pone.0109917

**Published:** 2014-10-08

**Authors:** P. Dilip Venugopal, Peter L. Coffey, Galen P. Dively, William O. Lamp

**Affiliations:** Department of Entomology, University of Maryland, College Park, Maryland, United States of America; Institute of Vegetables and Flowers, Chinese Academy of Agricultural Science, China

## Abstract

The local dispersal of polyphagous, mobile insects within agricultural systems impacts pest management. In the mid-Atlantic region of the United States, stink bugs, especially the invasive *Halyomorpha halys* (Stål 1855), contribute to economic losses across a range of cropping systems. Here, we characterized the density of stink bugs along the field edges of field corn and soybean at different study sites. Specifically, we examined the influence of adjacent managed and natural habitats on the density of stink bugs in corn and soybean fields at different distances along transects from the field edge. We also quantified damage to corn grain, and to soybean pods and seeds, and measured yield in relation to the observed stink bug densities at different distances from field edge. Highest density of stink bugs was limited to the edge of both corn and soybean fields. Fields adjacent to wooded, crop and building habitats harbored higher densities of stink bugs than those adjacent to open habitats. Damage to corn kernels and to soybean pods and seeds increased with stink bug density in plots and was highest at the field edges. Stink bug density was also negatively associated with yield per plant in soybean. The spatial pattern of stink bugs in both corn and soybeans, with significant edge effects, suggests the use of pest management strategies for crop placement in the landscape, as well as spatially targeted pest suppression within fields.

## Introduction

Agricultural fields are components within a heterogeneous landscape that strongly connect to and interact with surrounding habitats [1]. The movement of insects between natural and agricultural habitats has important implications for agricultural ecosystem function [2]. The movement of pest insects among seasonal crop resources is often non-random and directional as pest species disperse and colonize crops [3]. This movement may result in insect pests immigrating into an agricultural field in an aggregated manner in specific areas within the field [1]. In such cases, species-specific characteristics such as host range, vagility, chemical ecology, and host developmental status influence the spatial pattern of a pest population within crop fields, often resulting in the aggregation of pests along field edges as they disperse between habitats. The seasonal availability and suitability of source and recipient habitats in relation to the life stages of a mobile, polyphagous insect pest influence the dispersal dynamics of pests from sources to recipient habitats [2], [4], [5]. Knowledge about insect pest movement among habitats in the landscape, and the subsequent colonization of plants within crop fields, may inform risk of infestation by an insect pest prior to their subsequent population increase as well as provide opportunities for pest management [1].

Stink bugs in the family Pentatomidae are major pests of economically important crops globally [6], and are considered important pests in soybean *Glycine max* (L.) Merr. producing areas of the world [7]. While stink bugs caused economic losses in the southern United States, they were not considered serious pests of crops in the mid-Atlantic region until recently. The most common stink bugs in agricultural fields in the mid-Atlantic are *Chinavia hilaris* (Say 1832) and *Euschistus servus* (Say 1832), but these species have had little economic impact in the region [8], [9]. The recent explosion in populations of the invasive brown marmorated stink bug, *Halyomorpha halys* (Stål 1855) however, has led to significant economic and ecological impacts.

Since its discovery near Allentown, Pennsylvania, USA, *H. halys* has been detected in 41 states within United States, and local populations and detections from Europe (Switzerland, France, Canada, Germany, Italy, and Liechtenstein) have also been reported [10]. This polyphagous stink bug has a wide range of host plants including tree fruits, vegetables, field crops, ornamental plants, and native vegetation in its native and invaded ranges. Since 2010, serious crop losses have been reported for apples, peaches, sweet corn, peppers, tomatoes, and row crops such as field corn and soybeans in the mid-Atlantic region [11]. *Halyomorpha halys* is also a nuisance pest in human dwellings. In this context, information on the movement of stink bugs into crops from adjacent managed and natural habitats, and the associated spatially-explicit crop damage, has direct implications for integrated pest management.

The dispersal and movement of various stink bug species between crops and other habitats has been addressed by many studies in the context of dispersal between habitats, adjacent habitat influences on densities at field edges, and their relationship to crop damage [12]–[23]. However, these studies mainly pertain to stink bug communities in crops in the southern U.S. Currently only anecdotal reports of high *H. halys* abundance in the edges of fields adjacent to woodlots [11] are available. These reports are consistent with the documented use of woody trees as a food source and as overwintering sites by *H. halys* [24]. This factor coupled with use of buildings as overwintering sites suggests that these habitats, which support high local stink bug populations that eventually invade soybean later in the season, are likely to play an important role in stink bug densities.

Many stink bug species cause significant seed quality and yield losses in field corn, *Zea mays* L., and soybean [19], [23]–[29], and stink bugs are also associated with the transmission of plant diseases [32]–[34]. However, few studies quantify spatially-explicit field crop damage in relation to patterns of stink bug densities [35]. Soybean is one of the preferred hosts for *H. halys* [36], and both field corn and soybean constitute a very high proportion of overall crop area in the mid-Atlantic region and throughout the United States [37]. Research efforts aimed at determining the role of adjacent habitat in influencing stink bug dispersal, population density, and pattern of settlement into crop, contribute to the development of management strategies of *H. halys* in row crops.

In this study, our objectives were a) document the species composition and within-field distribution of stink bugs in field corn and soybean, b) examine the influence of adjacent managed and natural habitats on the density of stink bugs, and c) relate stink bug density to seed quality in field corn and soybean, and pod development and yield in soybean. We expected *H. halys* to be the most abundant stink bug in our study based on previous reports of stink species composition in mid-Atlantic row crops [8]. We predicted higher density of stink bugs along wooded habitats and buildings than open habitats as they provide host plants and over-wintering refuge [24]. We also predicted high density of stink bugs at the field edge, reducing with distance into the field interior as observed by anecdotal reports for *H. halys* [11].

## Methods

### Ethics statement

The field studies were conducted at facilities provided by the University of Maryland and the USDA Beltsville Agricultural Research Center where we had permission to conduct research. Stink bugs were enumerated *in situ* and were not collected, and no endangered or protected species were involved.

### Field selection and stink bug sampling strategy

The study was conducted at the USDA Beltsville Agricultural Research Center at Beltsville, MD (39°01′50N; 76°53′28W) and University of Maryland Research and Education Center facilities at Beltsville (39°00′45N; 76°49′32W), Clarksville (39°15′18N; 76°55′51W) and Keedysville, MD (39°30′37N; 77°43′57W). At these sites, field corn (76.2 cm row spacing) and full season soybean (17.8 cm row spacing) fields with a portion of their perimeter directly adjacent to wooded areas (henceforth wooded habitats), buildings (buildings, houses and barns; henceforth building habitats), crops (alfalfa, sorghum, and vegetable crops; henceforth crop habitats) and open, non-crop areas that were grassy, untilled land (henceforth open habitats) were sampled in 2012 and 2013. Corn fields were chosen as one of the adjacent habitat types *in lieu* of crops for several soybean fields. In each field, the sampling layout included four transects spaced 20 m apart. Each transect was marked for sampling plots at distances 0, 1.5, 3, 4.5, 6, 9, 12, and 15 m from the edge to field interior. Stink bugs were enumerated at each plot by carefully examining 10 consecutive corn plants and later converted to densities based on planting density, or all plants within a semicircular area of 0.5 m radius (1.57 m^2^) in soybean.

Visual counts of stink bug adults, nymphs, and egg masses of *H. halys*, *E. servus*, *C. hilaris*, *Murgantia histrionica* (Hahn 1834), and *Thyanta custator* (Fabricius 1803) were recorded and converted to densities. Fields and plots were sampled each week between mid-July and mid-August in field corn, and mid-August and late-September in soybean. Sampling coincided with the kernel development stages of corn (R2–R5; blister – dent; [38]) and the seed development stages of soybean (R4–R7; full pod to physiological maturity; [39]), which are associated with high *H. halys* and other stink bug species densities [8], [11], [40]. Details on the number of corn and soybean field edges with different adjacent habitats, and the sampling dates during 2012 and 2013 are provided in Table 1. Including the repeated sampling of fields, a total of 4,315 field corn plots in 31 fields, and 2,968 soybean plots in 26 fields across all sites were sampled for stink bugs during 2012 and 2013.

**Table 1:** Details on field corn and soybean field edges with different adjacent habitats sampled for stink bugs and the sampling occasions at each field in Maryland, USA during 2012**–**2013.

Crop	Year	Site	Adjacent habitats (number of field edges)	Sampling dates (frequency)
Field Corn	2012	Beltsville	woods (4), buildings (3), crops (1), open (4)	10 July–15 Aug (7–10 days)
		Clarksville	woods (1), buildings (1), crops (3)	10 July–15 Aug (7 days)
	2013	Clarksville	woods (3), buildings (2), crops (3), open (1)	18 July–22 Aug (7 days)
		Keedysville	woods (1), buildings (1), crops (1), open (2)	16 July–20 Aug (7 days)
		Overall	woods (9), buildings (7), crops (8), open (7)	10 July–22 Aug (7–10 days)
Soybean	2012	Beltsville	woods (2), buildings (3), corn (1), open (2)	23 Aug–20 Sept (7–10 days)
		Keedysville	woods (2), buildings (1), corn (2), open (1)	30 Aug–26 Sept (7–10 days)
	2013	Beltsville	woods (1), buildings (1), corn (1), open (1)	13 Aug–06 Sept (7 days)
		Clarksville	buildings (1), corn (1), open (1)	16 Aug–12 Sept (5–7 days)
		Keedysville	woods (2), buildings (1), corn (1), open (1)	15 Aug–18 Sept (5–7 days)
		Overall	woods (7), buildings (7), corn (6), open (6)	13 Aug–26 Sept (5–10 days)

### Assessing seed quality in field corn and soybean

To relate stink bug density to ear damage in corn, eight fields with the highest observed count of stink bugs in 2013 and with different adjacent habitats were selected. Of these fields, 3, 3, 1 and 1 were adjacent to wooded, building, crops and open habitats, respectively. In each field, 6–10 consecutive corn ears were collected at each sampling plot prior to harvest maturity and stored in cloth bags for drying. Planting details of the fields used for assessing corn damage are provided in Table 2. For each ear, the following data were recorded once at the end of the season: 1) number of kernels damaged by stink bugs (identified by a characteristic puncture scar typically surrounded by a discolored cloudy marking); 2) number of collapsed kernels due to stink bug damage (this type of damage was carefully examined to distinguish between kernels damaged by stink bugs and dusky sap beetles, *Carcophilus lugubris* Murray); 3) number of kernel rows around the ear; 4) length of one kernel row (mm); and 5) average width of individual kernels (mm). With the individual ear measurements, the total number of kernels was derived by dividing the kernel row length by the width of a kernel times the number of rows. Data were then summed across all ears of a plot and stink bug damage was expressed as the percentage of damaged and collapsed kernels in relation to total number of kernels per sample. A total of 2,326 ears of corn from 252 sampling plots across 8 fields were assessed for stink bug damage.

**Table 2:** Details on the field corn and soybean fields used for analyzing grain and seed damage in Maryland, USA during 2012–2013.

Crop	Year	Site	Field ID	Variety	Planting Date	Density/acre
Field Corn	2013	Clarksville	Corn1	P1319HR (113)	2May13	26,000
			Corn2	DKC61-21 (111)	15May13	26,000
			Corn3	P1319HR (113)	2May13	26,000
			Corn4	P1319HR (113)	2May13	26,000
			Corn5	DKC61-21 (111)	16May13	26,000
			Corn6	NK74R3000GT (114)	16May13	26,000
		Keedysville	Corn7	Doebler’s 633HXR (110)	23Apr13	26,000
			Corn8	Doebler’s 633HXR (110)	23Apr13	26,000
Soybean	2012	Beltsville	Soy1	AG3030 (3.0)	11May12	155,555
			Soy2	AG3030 (3.0)	11May12	155,555
		Keedysville	Soy3	Doebler 3809RR (3.8)	26May12	180,000
			Soy4	Doebler 3809RR (3.8)	4Jun12	180,000
			Soy5	Doebler 3809RR (3.8)	4Jun12	180,000
	2013		Soy6	SCS9360RR (3.5)	22May13	180,000
			Soy7	Doebler 3809RR (3.8)	27May13	180,000

For each of the corn and soybean varieties, the corn relative maturity in days and the soybean maturity groups respectively, have been provided in parenthesis.

To relate stink bug density to soybean pod development prior to harvest, samples of 10 consecutive plants at each plot of seven soybean fields in 2012–2013 were examined once at the end of the season *in situ* to count the total numbers of pods with 3 or more fully developed seeds (full pods), pods with fewer than 3 seeds (half pods), and flat, immature pods (flat pods). For standardization, the proportions for each pod type were calculated for each sample. Pod quality data were collected from 64 plots in 2 fields adjacent to wooded habitat at Keedysville. Planting details for the fields used for assessing soybean seed damage are provided in Table 2. Of the 7 fields sampled for seed quality data, 2 were adjacent to buildings, and 5 fields were adjacent to wooded habitats, and all had the highest counts of stink bugs observed in each year. Just prior to harvest, 20 plants each from 154 plots in the fields were collected, stored in mesh bags, and allowed further drying for optimal thrashing. Seeds were removed from pods for each sample by a stationary motor-driven thrasher (RL Brownfield-Swanson Machine Company; Model B1). Dirt, chaff, or un-thrashed pods were removed, and the remaining seed samples were then weighed to measure yield (measured as grams/20 plants).

To assess seed quality, subsamples of 200–300 seeds were removed from each sample, counted, and weighed to calculate test weight (expressed as weight per 100 seeds). Seed samples in 2012 were sieved to separate smaller, immature seeds (<0.3 cm), whereas these smaller seeds were not removed from subsamples in 2013. In both years, seeds were individually examined and categorized into six groups as follows: 1) stink bug damaged seed, distinguished by a puncture scar and often surrounded by a discolored cloudy area; 2) moldy seed, characterized by having milky white or grayish crusty growth on surface, sometimes with cracks and fissures; 3) shriveled seed that appeared wrinkled and often undersized; 4) purple seed recognized as purple or pink areas on the seed coat due to the fungus *Cercospora kikuchii* Matsumoto & Tomoy 1925 [41]; 5) green seed showing discolored green tissue in cross section, rather than the normal yellow; and 6) normal, undamaged seed. Seeds with characteristics of more than one damage category were assigned to whichever category seemed dominant, except for stink bug damaged seeds which were all assigned to that category. To standardize across samples, the percentage of seeds in each category was calculated. Soybean seed quality data were collected for a total of 154 plots from 4 fields in 2012 and 2 fields in 2013.

### Statistical analyses

#### Adjacent habitat and distance from edge influences

The influence of adjacent habitat and distance from field edge on the density and distribution of stink bugs was analyzed by Generalized Linear Mixed Models (GLMMs) based on Laplace approximation, with a Poisson-lognormal error distribution and log link function [42]. All analyses were performed with fields as replicates and the sampling plots along transects within the field as subsamples. For corn and soybean data, analyses were performed on –data pooled over all stink bug life stages. For each of these datasets, GLMMs were performed on the data pooled across species, years, and study sites, and on data from each study site pooled across years. Sampling points within each field edge was treated as a random factor to control for repeated measurement [43]; adjacent habitat, distance from edge, and their interaction were the fixed effects, and stink bug density was the response variable. For the overall data models, study site and year were also treated as random effects.

Model building and selection procedures for the mixed effects modeling followed the procedures used by Zuur and others [44]. First, several candidate models, each with different random effects but identical fixed effects, were tested to choose the optimal random effect model using a combination of AIC and BIC values for selection criteria [36]. For all optimum fixed effect models, an initial full model analysis including individual and interactive effects of adjacent habitat (4 levels - woods, buildings, mix crop/corn, and open) and distance from edge was performed. Specifically we designed a model matrix that directly estimated the intercepts and the slopes for each level of adjacent habitat (extension of means parameterization; see pages 61–64 in [45]) in relation to distance from field edge. The significance of the fixed effects was determined by Wald χ^2^ tests. If a significant interaction was found these estimates, directly interpreted as the intercept and slope of the regression of stink bug density on distance for each of the adjacent habitat types, were then simultaneously compared through post-hoc comparisons with a Bonferroni correction. Models were evaluated for assumption appropriateness by testing for over-dispersion and correlations among random effect terms, and by visualizing variances in a location-scale plot with superimposed loess fit [42].

#### Relating stink bug density and seed damage

Influence of stink bug density on damage to corn kernels was assessed using generalized linear models (GLMs) with Poisson or quasi-Poisson error distribution and log link function [46]. Percentage of collapsed and stink bug damaged seeds were used as the response variables and mean stink bug density, the explanatory variable. For significant results, the coefficient of determination was calculated by Nagalkerke’s pseudo r^2^ statistic [47].

Linear regression was used to assess the influence of stink bug density on soybean pod development. To meet normality assumptions, response variables (% flat or full pods) was square root transformed prior to analysis. Influence of stink bug density on soybean seed quality was assessed by linear mixed models (LMMs) with year as a random effect to account for minor differences in grading seed size protocols. LMMs related stink bug density to the percentage of seeds in each category of seed quality. Influence of stink bug density on soybean yield per plot was assessed by LMMs with field as a random effect to account for differences in soybean variety and other field conditions among sites. Response variables (% seeds in various damage categories, or yield) were log or square root transformed to meet normality requirements and the significance of the fixed effect was determined by Wald t-tests. Diagnostic plots of the models visualizing within-group residuals (standardized residuals vs fitted values, normal Q-Q plots, and histograms of residuals) and estimated random effects (normal Q-Q plots and pairs-scatter plot matrix) were used to assess model appropriateness. The coefficients of determination for the LMMs, based on the likelihood-ratio test, were calculated using Nagalkerke’s pseudo r^2^ statistic [47]. Patterns in damage to corn kernels, soybean pods, and seeds at different distances from the edge, in relation to stink bug density, were visualized by plotting average values of damage and stink bug density aggregated by distance.

All statistical analyses were performed in R program [48] and associated statistical packages. GLMMs were performed with package ‘lme4’ [49] and LMMs with package ‘nlme’ [50]. Multiple comparisons of means for GLMMs were computed with R packages ‘contrast’ [51] and ‘multcomp’ [52]. GLMMs and LMMs estimated coefficients were extracted through package ‘effects’ [53], converted to densities/m^2^ and plotted using ‘ggplot2’ [54]. Coefficient of determination (pseudo r^2^) for the GLM was calculated with package ‘rms’ [55], and with package ‘MuMin’ [56] for LMMs.

## Results

### Species composition and density

A total of 9440 individuals (66% nymphs; 34% adults) of four phytophagous stink bug species (*E servus*, *H. halys*, *C. hilaris*, and *M. histrionica*) were recorded in field corn, of which *H. halys* accounted for 97% of the total. Species composition varied among study sites and crop systems. *Halyomorpha halys* comprised 57% of the sampled populations in corn at Beltsville, followed by *E. servus* (35%), whereas *H. halys* accounted for ∼97% of all stink bugs at Clarksville and Keedysville. In soybean, a total of 9867 individuals (68% nymphs; 32% adults) of five phytophagous stink bug species (*E. servus, H. halys, C. hilaris, M. histrionic, T. custator*) were recorded, of which *H. halys* accounted for 93% of the total. *Halyomorpha halys* comprised 83–85% of the stink bug numbers in soybean at Beltsville, while greater than 92% were *H. halys* at Clarksville and Keedysville. Results obtained from the statistical analyses hence pertain mainly to patterns of *H. halys* density, since this species constituted ∼95% of all observed stink bugs in both field corn and soybean.

### Adjacent habitat and distance from field edge influences

The trends for density among adjacent habitats at different distances from the edge observed in adult and nymph data sets were broadly similar and were hence pooled for the analyses, and we report only these results.

#### Field corn

**Figure 1:**
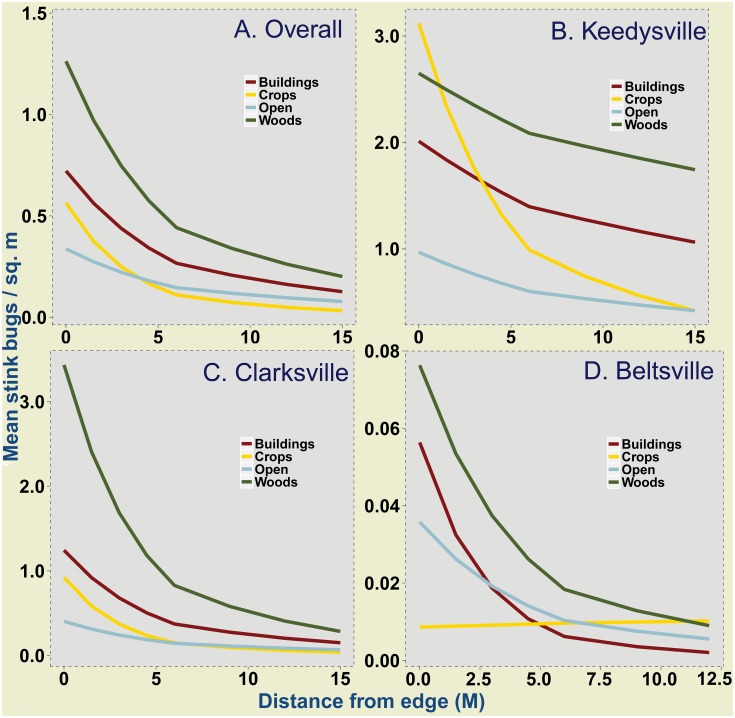
Mean stink bug density in field corn in relation to different adjacent habitats and distance from the field edge. Estimates derived from Poisson-lognormal GLMMs are plotted for overall stink bug data pooled over all study sites (A), Keedysville (B), Clarksville (C) and Beltsville (D). Values presented here have been back transformed from their original link function estimated model coefficients. Multiple comparison of means with a bonferroni correction (α = 0.05) showed significant differences in: overall (A) - wooded habitats and buildings and, wooded and open habitats; Keedysville (B) – all adjacent habitats significantly different from each other; and Clarksville (C) - wooded habitats and buildings and, wooded and open habitats. For Beltsville (D), all multiple means comparisons were non-significant.

For the analysis of overall stink bug data from field corn edges, the random effects used for the GLMM included the field, study site and year. Results showed significant interactive influences of adjacent habitat and distance from edge on stink bug density (Wald χ^2^ = 492.6, DF = 8, *P*<0.001). Simultaneous comparison of intercepts and slopes among adjacent habitats accounting for the influence of distance from edge revealed significant differences. Overall, the density of stink bugs at different distance from the edge was significantly higher in fields adjacent to wooded habitats compared to density in fields adjacent to buildings and open habitats (Fig. 1A). Density between wooded and crop habitats did not significantly differ, although mean densities of stink bugs were consistently higher adjacent to wooded habitats (see Fig. S1 for variance around estimated means). Similarly stink bug density did not differ significantly between crop habitats and buildings although higher mean density was observed along buildings.

For site level GLMMs, significant interactive influences of adjacent habitat and distance from edge on stink bug density were observed for all the sites - Clarksville (Wald χ^2^ = 426.9, DF = 8, *P*<0.001), Keedysville (Wald χ^2^ = 367.9, DF = 8, *P*<0.001) and Beltsville (Wald χ^2^ = 43.1, DF = 3, *P<*0.001). Multiple comparisons of means of stink bug density at Clarksville showed similar trends to the results of analyses of data pooled over all study sites, with density in fields adjacent to wooded habitats higher than density adjacent to buildings and open habitats (Fig. 1C). Similar to overall data, at Clarksville, stink bug density did not differ significantly between wooded and crop habitats, although mean densities of stink bugs were consistently higher adjacent to wooded habitats. Similarly stink bug density did not differ significantly between crop habitats and buildings although higher mean density was observed along buildings. At Keedysville however, stink bug density was highest along fields adjacent to crops, and the slopes and intercepts were significantly different among adjacent habitats (Fig. 1B). Additionally, corn fields at Keedysville had higher stink bug density along the outside rows (0 m) adjacent to crops than adjacent to woods. At Beltsville, with the least stink bug density, multiple comparisons did not reveal significant differences in stink bug density among adjacent habitats, after accounting for the distance from edge influence (Fig. 1D; also see Fig. S1 for variance around estimated means for each site). The raw means for each site and year (Fig. S2) are provided as supplementary information.

#### Soybean

**Figure 2:**
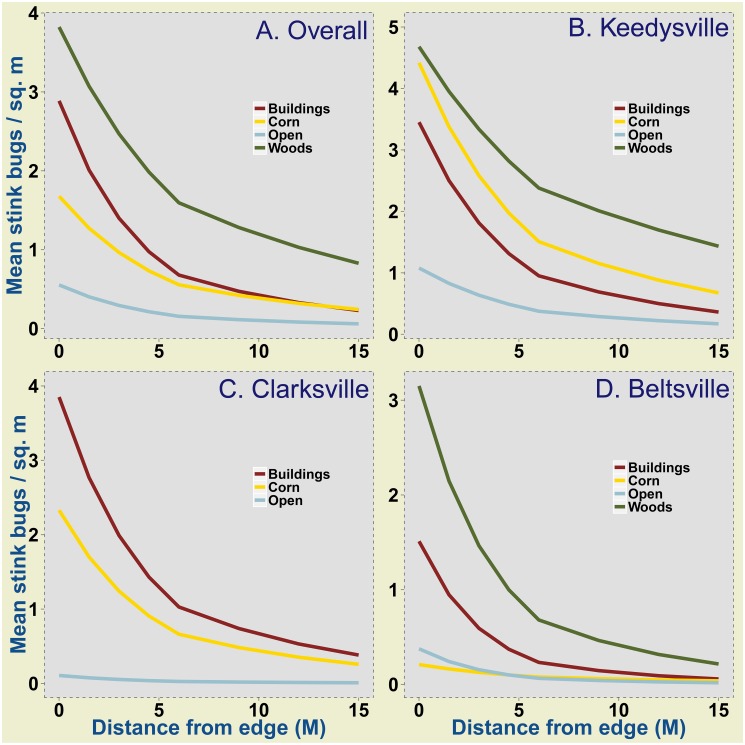
Mean stink bug density in soybean field edges in relation to different adjacent habitats and distance from field edge. Estimates derived from Poisson-lognormal GLMMs are plotted for overall stink bug data pooled over all study sites (A), Keedysville (B), Clarksville (C) and Beltsville (D). Values presented here have been back transformed from its original link function estimated model coefficients. Multiple comparison of means with a bonferroni correction (α = 0.05) showed significant differences in: overall (A) - wooded habitats and buildings, wooded and open habitats, buildings and corn habitats, buildings and open habitats; Keedysville (B) – wooded habitats and buildings, wooded and open habitats, buildings and corn habitats, buildings and open habitat; Clarksville (C) - buildings and open habitats, and corn and open habitats; Beltsville (D) – buildings and open habitats, buildings and wooded habitats, and wooded and corn habitats.

The GLMM analysis of overall stink bug data treated field as the only random effect and showed significant interactive influences of adjacent habitat and distance on stink bug density (Wald χ^2^ = 717.2, DF = 8, *P*<0.001). Multiple comparisons of means showed that density of stink bugs was significantly higher at all distances in fields adjacent to wooded habitats compared to density in fields adjacent to buildings and open habitats (Fig. 2A). Stink bug density adjacent to wooded habitats was also consistently higher than density in fields adjacent to corn field habitats, but differences were not significant (see Fig. S3 for variances around estimated means). Across all the adjacent habitat types the highest density of stink bugs was recorded at the immediate field edge (0 m), and was lowest at 15 m from edge (<1/m^2^).

GLMMs performed by study site on overall stink bug data showed significant interactive influence of adjacent habitat and distance from edge on density at all the sites - Keedysville (Wald χ^2^ = 485.4, DF = 8, *P*<0.001), Clarksville (Wald χ^2^ = 216.63, DF = 5, *P*<0.001) and Beltsville (Wald χ^2^ = 279.2, DF = 8, *P*<0.001). Multiple means comparisons for Keedysville data showed significantly higher densities adjacent to wooded habitats buildings and open habitats (Fig. 2B). The intercept and slopes for buildings and corn, and buildings and open areas were also significantly different. At Clarksville, buildings and corn habitats had significantly higher density of stink bugs than open areas (Fig. 2C). Model estimated stink bug densities for buildings and corn habitats were not significantly different. At Beltsville, wooded habitats and buildings had significantly higher stink bug density than corn as adjacent habitat (Fig. 2D). The slope and intercept were significantly different for wooded habitats and buildings. Unlike the other sites where stink bugs were fewest adjacent to open habitats, at Beltsville, corn as adjacent habitat harbored the lowest density of stink bugs. The variance around the estimated density of stink bugs in soybean fields for each site (Fig. S3), and the raw means for each site and year (Fig. S4) are provided as supplementary information.

### Corn and soybean seed damage

For field corn, results from the quasi-Poisson GLM showed a significant positive association between percentage of stink bug damaged kernels and mean stink bug density (y = 0.57+0.15x, n = 252, *P*<0.001, pseudo r^2^ = 0.47). A Poisson GLM showed that the percentage of collapsed kernels was not significantly associated with mean stink bug density (y = −6.75+0.14x, n = 252, *P* = 0.50, pseudo r^2^ = 0.17). For soybean pod development data, regression analysis revealed that the percentage of full pods was negatively influenced by mean stink bug density (y = 5.9−0.17x, n = 63, *P*<0.001, r^2^ = 0.51), while percentage of flat pods (square root transformed) was positively influenced (y = 2.18+0.26x, n = 63, *P*<0.001, r^2^ = 0.63). Results of LMMs analyzing each seed quality category (Table 3) showed a significant positive association of mean stink bug density and purple stained seeds, percentage of stink bug damaged seeds, percentage of immature, shriveled and moldy seeds, and overall percentage of damaged seeds.

**Table 3:** Statistical results of LMMs for analyzing the relationship between stink bug density and various soybean seed damage categories and yield.

Response variable	Data Transformation	Intercept	Intercept SE	Estimate	SE	DF	Wald t	Pval	psuedo r^2^
% normal seeds	None	75.8	8.04	−2.11	0.25	145	8.28	<0.001	0.30
% stink bug damaged seeds	Square Root	3.41	0.5	0.07	0.01	145	4.58	<0.001	0.12
% purple damaged seeds	log	1.39	0.14	0.09	0.01	145	9.99	<0.001	0.44
% moldy + shriveled + immature seeds	Square Root	2.59	0.47	0.09	0.02	148	5.87	<0.001	0.19
% all damaged seeds	Square Root	4.78	0.63	0.18	0.02	145	9.03	<0.001	0.35
Total Yield (grams/20 plants)	Square Root	17.1	1.04	−0.20	0.04	140	4.67	<0.001	0.13

**Figure 3:**
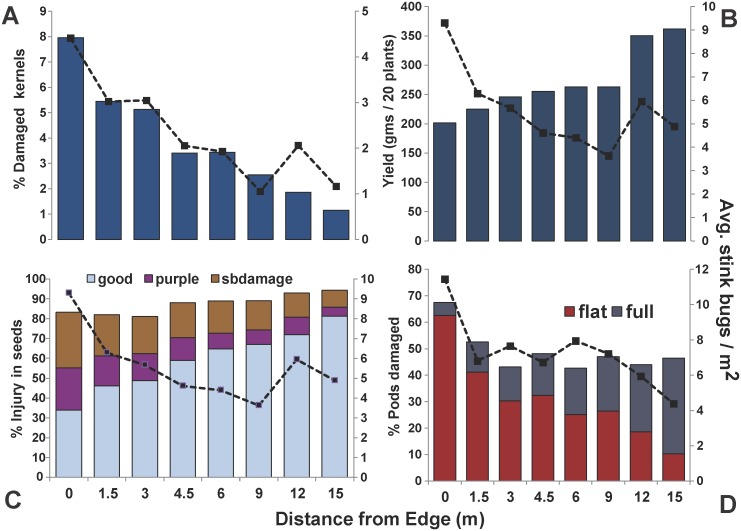
Patterns of kernel damage in field corn (A), soybean yield (B), soybean seed damage by category (C), and soybean pod development (D) in relation to mean stink bug density at different distance from field edge. The proportions of soybean seeds in each seed quality category (stink bug damaged, purple damaged, and normal seeds) and pod types (flat and full) are also provided. Mean stink bug abundance are denoted by the dashed lines which represent the second y axis.

Overall percentage of normal, undamaged soybean seeds and yield had a significant negative relationship with stink bug density (Table 3). The overall seed damage by stink bugs in both corn (Fig. 3A) and soybean (Fig. 3C), and their impact on soybean pod development (Fig. 3D), were highest at field edge and declined gradually towards the field interior. Furthermore, soybean yields (grams/20 plants) were lowest at field edge, gradually increasing from field edge to highest yields at 12 and 15 m from the edge (Fig. 3B).

## Discussion

Seasonal availability and suitability of source and recipient habitats influence dispersal dynamics of pest populations [2], [4], [5]. Aggregation of stink bugs at the field edge of target crops results from their directional movement among a sequence of hosts in an area in response to deteriorating suitability of the host crops or native vegetation and increasing suitability of the target crop plants [57]. Our results provide strong evidence that *H. halys* density exhibits a clear edge effect in both field corn and soybean. Across all adjacent habitats, density of *H. halys* and other stink bugs was highest within the first 3 meters, reaching lowest levels between 9–15 m from the edge, least at 15 m from the edge (<1/m^2^). In addition to host suitability, species specific behaviors contribute to the resulting aggregations of stink bugs along crop edges. The strong edge effect exhibited by *H. halys* is similar to the within-field infestation pattern reported for other native stink bug species in U.S. crops [12], [15], [17]–[19], [23], [58]. We demonstrated that the aggregations are associated with specific kinds of damage to both corn grain and soybean seeds, thus suggesting that sitenspecific management at the field and farmscape levels may improve crop protection from stink bugs.

The timing of infestations during mid to late July in corn, and then later colonization of soybean fields in August, suggests that the majority of *H. halys* adults were offspring of the generation which developed on host plants earlier in the season (June/July). *Halyomorpha halys* is known to feed on a wide range of cultivated and wild hosts (up to 170 species) [59], of which many tree and shrub species are likely present in wooded habitats. Particularly high density of *H. halys* was observed (personal field observations, Dilip Venugopal) in soybean fields bordering wooded habitats with tree of heaven (*Ailanthus altissima* Swingle), princess tree (*Paulownia tomentosa* Baill.), and black cherry (*Prunus serotina* Ehrhart), all which support high population densities of reproducing *H. halys* [59], [60]. Similarly, earlier reports show that *C. hilaris* populations in soybean are greater adjacent to wooded borders with black cherry and elderberry (*Sambucus canadensis* L.) [13]. Additionally, *E. servus* populations are greater in cotton fields with adjacent woods containing many oak species (*Quercus* spp.) and black cherry [18].

This study also addressed the influence of adjacent habitats on stink bug density and quantified differences in density at various distances from the field edges in soybean and corn crops. We found that adjacent habitats, particularly wooded habitats, influenced the densities of *H. halys* and other stink bugs. In both corn and soybeans, fields adjacent to wooded habitats, across all study sites and distances from field edge, consistently harbored significantly higher densities of stink bugs than in fields adjacent to open habitats. Also, stink bug density in fields adjacent to wooded habitats was consistently higher than in fields adjacent to buildings and corn habitats. These results suggest that wooded habitats play an important role in serving as sources of stink bug populations that colonize crops.

Patterns of infestation along field edges of corn and soybean differed among study sites, partly influenced by overall stink bug density and other adjacent habitats. Stink bug density in both row crops at Keedysville was consistently (3–5 times) greater than the mean density at the other two study sites, and this could be attributed to the higher overall densities of *H. halys* observed in the Western Maryland region over the past 5 years. In addition to wooded habitats, building and other crop habitats served as sources of colonizing adults in row crops. *H. halys* often utilize buildings as overwintering sites and thus these structures would likely influence stink bug populations earlier in the spring when post-diapause adults are moving to host plants, thereby supporting high local populations that eventually invade soybean later in the season. At Keedysville, stink bug density was higher in corn fields adjacent to alfalfa and in soybean fields adjacent to corn than in corn or soybean fields adjacent to building habitats. However, at Clarksville and Beltsville adjacent building habitats showed a higher density of stink bugs than crop habitats.

As with other studies, our results highlight the role of other adjacent cultivated crops in influencing stink bug populations. For example, adjacent fields of alfalfa, field corn, and other cultivated borders have been reported as a sources contributing to higher densities of stink bugs in tomato, cotton, sorghum, and peanut fields [12], [16], [19], [23]. However, differences in the relative influence of adjacent habitats in our study could be related to overall stink bug density at each of the study sites. For example, stink bug density did not differ significantly among adjacent habitats in field corn at Beltsville where *H. halys* densities were lowest. Moreover, the influence of the landscape heterogeneity on stink bug density could extend to larger spatial scales beyond habitats adjacent to a crop. Since insect population dynamics and spatial patterns are affected by regional landscape context and species traits such as dispersal ability [61], distribution, and density of *H. halys* may depend on habitat and other environmental characteristics at spatial scales greater than the local agricultural field [62], [63]. Differences in landscape structure among our study sites may explain the higher density of stink bugs at Keedysville, and specifically the role of adjacent crop habitats as sources of stink bugs in field corn and soybean.

We related the various corn and soybean damage measurements to stink bug density within plots. As expected, stink bug damage to corn kernels increased with stink bug density. The percentage of damaged kernels reached levels up to 8% at the field edge to less than 3% between 9–15 m from field edge, and was positively correlated with the stink bug density within plots. The percentage of collapsed kernels was negligible and not significantly influenced by stink bug density. Based on findings by earlier studies [25], [64], neither kernel damage, ear weight or grain weight are affected beyond tasseling stage (VT) from feeding damage by *E. servus* or *N. viridula*. Although *H. halys* density can be high along edges of corn fields, our result suggests that *H. halys* kernel quality loss is restricted to about 10 m from the field edge.

*Halyomorpha halys* populations in soybean had a significant impact on pod development, with the percentage of flat pods significantly increasing with increasing stink bug density. Concomitantly, the proportion of fully developed pods significantly decreased with increasing stink bug density. Changes in the development and maturation of soybean pods due to *H. halys* feeding have been recently documented [35], showing that most severe pod loss occurred at the R4 (full pod) growth stage. Observed effects on pod and seed development with higher stink bug density were similar to damage caused by other stink bug species [26]–[31]. Our finding that higher stink bug density is related to increased proportions of moldy and purple stained seeds suggest a potential role of *H. halys* in transmitting or facilitating various pathogens; however, this needs to be further investigated experimentally. Our study found a significant, yet weak negative association between soybean yield and stink bug density per plot. In contrast, recent field research using cages addressed the effects of *H. halys* feeding on soybean growth, and did not detect a significant relationship between H. *halys* stink bug densities and yield loss [35]. Soybean field studies to compare yields of insecticide-treated and untreated plots are needed to establish the relationship between soybean yield losses and stink bug density.

Knowledge of how adjacent habitats influence *H. halys* populations and the within-field distribution has several implications in stink bug management. First, our results indicate that scouting corn and soybean fields for decision-making is more efficient if efforts initially concentrated on field edges bordering wooded habitats where there is a greater likelihood of colonization and higher risk of infestation. Secondly, the infestation patterns of stink bug communities dominated by *H. halys* are primarily edge-centric, and population densities beyond 12 m are generally low (<1/m^2^). Based on our results, if insecticides are to be applied, edge-only applications, particularly along wooded and building habitats, could reduce the cost of control while preventing damage caused by stink bugs in field corn and soybean. Preliminary studies show that treating just 12 m into soybean field prevented further invasion by *H. halys* and other stink bugs (Ames Herbert, personal communication). The edge-only treatment prevented reinvasion and also resulted in an 85–95% reduction in insecticide used compared with whole-field treatments [11]. Results presented here showing highest stink bug density and associated damage limited to the immediate field edge provide validity for the edge-only treatment. While experimental research on effective insecticide application strategies are currently underway, based on our findings, we suggest that integrated pest management programs for the stink bug complex in field crops should include farmscape-level planning - crop location with regards to adjacent habitats, and targeted interventions in the form of edge-only treatments to prevent seed quality and yield losses.

## Supporting Information

Figure S1 **GLMM estimated mean stink bug densities in field corn (bold lines) and 95% CI (shaded region) among adjacent habitats and distance from the field edge.**
(TIF)

Figure S2 **Site and year wise raw stink bug averages in field corn among adjacent habitats and distance from the field edge.**
(TIF)

Figure S3 **GLMM estimated mean stink bug densities in soybean (bold lines) and 95% CI (shaded region) among adjacent habitats and distance from the field edge.**
(TIF)

Figure S4 **Site and year wise raw stink bug averages in soybean among adjacent habitats and distance from the field edge.**
(TIF)
