# Supplementary figures and images for: Adjacent Habitat Influence on Stink Bug (Hemiptera: Pentatomidae) Densities and the Associated Damage at Field Corn and Soybean Edges
Source: PLoS One. 2014 Oct 8;9(10):e109917. doi: 10.1371/journal.pone.0109917 (PMC4190369; doi:10.1371/journal.pone.0109917)

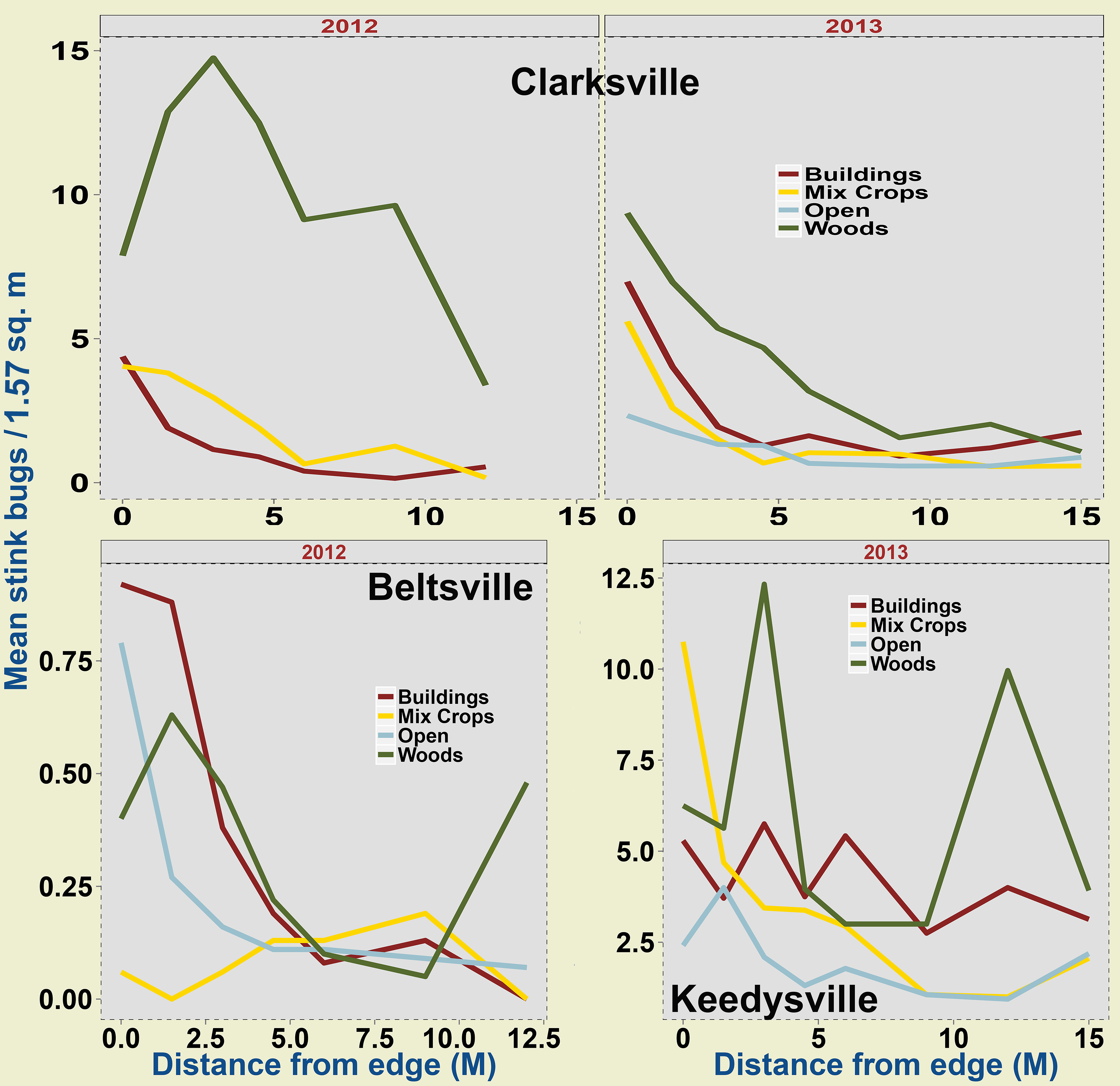

Supplement: Figure S2 — Site and year wise raw stink bug averages in field corn among adjacent habitats and distance from the field edge. (TIF) [file pone.0109917.s002.tif]

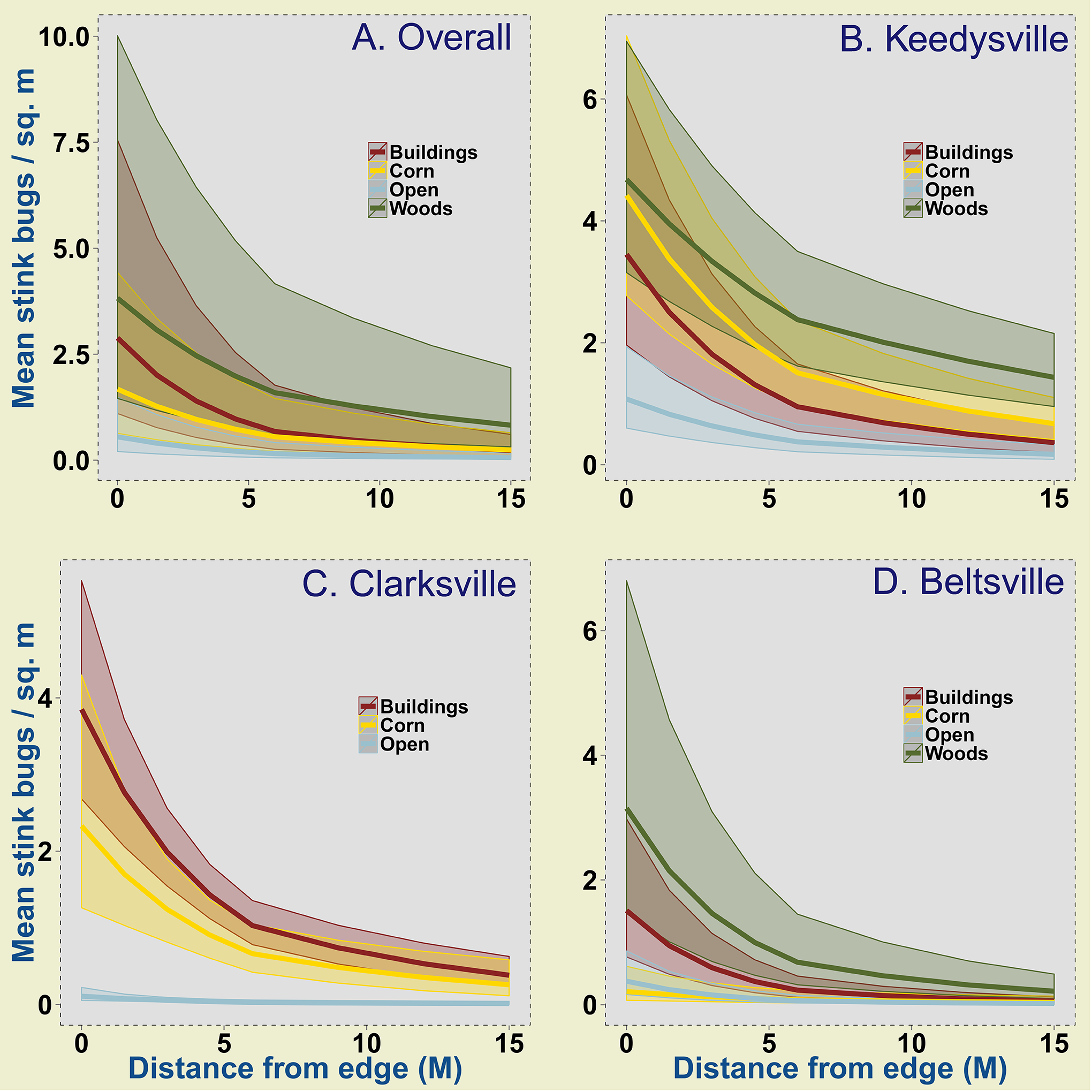

Supplement: Figure S3 — GLMM estimated mean stink bug densities in soybean (bold lines) and 95% CI (shaded region) among adjacent habitats and distance from the field edge. (TIF) [file pone.0109917.s003.tif]

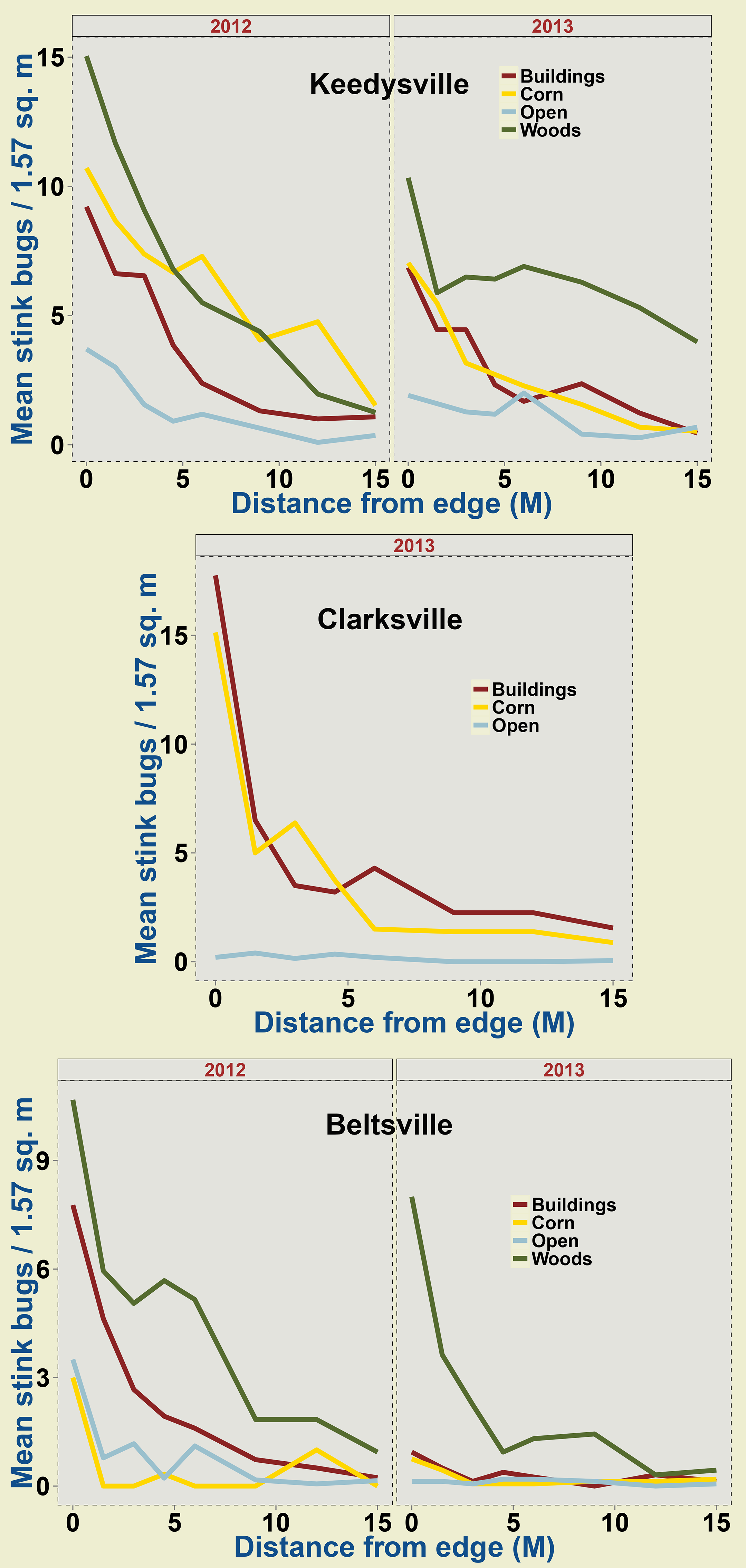

Supplement: Figure S4 — Site and year wise raw stink bug averages in soybean among adjacent habitats and distance from the field edge. (TIF) [file pone.0109917.s004.tif]
